# Evaluating the Impact of Adjustable- and Fixed-Loop Femoral Endobuttons on Intraoperative Surgeon Satisfaction and Postoperative Patient Functional Outcomes in Anterior Cruciate Ligament (ACL) Reconstruction

**DOI:** 10.7759/cureus.73411

**Published:** 2024-11-10

**Authors:** Nego Zion, Tapan K Das, Jitendra Mishra, Spandan Mishra, Shishir Agnihotri

**Affiliations:** 1 Department of Orthopedics, Institute of Medical Sciences and SUM Hospital, Bhubaneswar, IND

**Keywords:** acl tear, adjustable loop, arthroscopy, fixed-loop endobutton, knee injuries, surgeon satisfaction

## Abstract

Introduction: This study aims to evaluate the effects of adjustable- and fixed-loop femoral endobuttons on intraoperative surgeon satisfaction and postoperative patient functional outcomes in anterior cruciate ligament (ACL) reconstruction. The use of cortical suspensory devices, either fixed-loop or adjustable-loop, is common in ACL reconstruction surgeries for femoral tunnel fixation. Fixed-loop devices, although effective, often require additional tunnel drilling, potentially leading to tunnel widening. Adjustable-loop devices were introduced to mitigate this issue. However, there are concerns regarding the mechanical performance of adjustable loops, particularly under cyclical loading, which could affect long-term graft stability.

Materials and methods: This retrospective comparative study compares functional outcomes and intraoperative assessments of 140 patients who underwent ACL reconstruction between 2017 and 2021 with fixed-loop and adjustable-loop devices for the femoral side. Patients were evaluated at six weeks, six months, one year, and two years postoperatively using the Lysholm score, International Knee Document Committee score, Tegner activity scores, and knee stability tests, including Lachman and anterior drawer tests. Additionally, the study captured surgeon satisfaction intraoperatively and compared it with the final functional outcome.

Results: Both groups demonstrated significant improvements in knee stability, range of motion (ROM), and functional scores over time. At six weeks, the anterior drawer test showed 61 stable knees out of 70 in the adjustable-loop group and 57 of 70 in the fixed-loop group, with stability maintained in 56 and 49 knees, respectively, at the final follow-up. The mean extension at two years was 0.5° ± 3.8° in the fixed-loop group and -1.3° ± 3.2° in the adjustable-loop group (p = 0.02). Although the adjustable-loop group showed trends toward better early knee stability and extension, these differences were not statistically significant at two years (p > 0.05).

Conclusion: Surgeons' intraoperative satisfaction with graft firmness was higher in the adjustable-loop group. However, this did not correlate with long-term functional outcomes, as both groups had comparable scores at the final follow-up. Further research with larger sample sizes and extended follow-up periods is needed to confirm these findings. The limitations of this study include its retrospective design, the potential for selection bias, and the subjective nature of intraoperative assessments.

## Introduction

Arthroscopic anterior cruciate ligament (ACL) reconstruction is a widely performed procedure to restore knee stability following ACL tears, commonly occurring during high-impact sports or physical activities. Femoral fixation is a critical component of this surgery, and various cortical suspensory devices are available for this purpose. In the early stages, fixed-loop devices were the primary choice for ACL reconstruction. However, they require additional tunnel drilling to accommodate button flipping, which can create tunnel dead space and potentially lead to femoral tunnel widening over time [1]. Misalignments during drilling may also result in a "bungee cord effect," compromising graft stability and effectiveness [2].

An upgraded option, adjustable-loop devices, was introduced to address the limitations associated with fixed-loop devices. Adjustable loops offer advantages such as reduced femoral tunnel widening, enhanced graft healing, minimized micromovement, and decreased bone removal, all of which improve postoperative outcomes [3-6]. However, despite these advancements, biomechanical studies indicate that adjustable loops may have inferior load resistance compared to fixed loops and can experience lengthening during cyclical loading, which raises concerns regarding their long-term performance [7,8]. Prior studies comparing these devices have found no significant differences in patient-reported functional outcomes, though they call for further research to confirm these findings and support clinical decision-making [9].

In ACL reconstruction, achieving optimal knee stability and functional outcomes is essential for patient recovery and long-term mobility. Although sometimes subtle, variations between fixed-loop and adjustable-loop devices may have important implications for recovery experiences and outcomes. For instance, adjustable loops that provide greater intraoperative stability may reduce micromovements, potentially enhancing graft healing and stability under load. Additionally, differences in knee extension and flexion may impact a patient's capacity to resume prior activity levels, influencing quality of life and reinjury risk. By investigating these potential differences, this study aims to offer insights to help clinicians make device choices to better align with patient-centered outcomes in ACL reconstruction. Exploring the functional outcome differences between these two devices is particularly relevant in India, where there is a demand for cost-effective implants. Although prior studies have compared fixed- and adjustable-loop devices in ACL reconstruction, findings remain inconclusive regarding their long-term functional outcomes and mechanical stability under cyclical load. Most existing studies have primarily focused on immediate postoperative stability, with fewer examining device performance over extended follow-up periods.

In orthopedic practice, feedback mechanisms, including intraoperative surgeon satisfaction, are often believed to correlate with long-term patient outcomes. However, existing studies, such as one by Harris et al., suggest that surgeons may overestimate recovery outcomes in trauma patients compared to patient-reported experiences [10]. While patient-reported outcomes (PROs), like International Knee Document Committee (IKDC) scores, are standard measures in ACL reconstruction evaluations [11], there is limited research exploring the relationship between intraoperative surgeon satisfaction with graft firmness and final patient functional outcomes.

This study aims to fill this gap by comparing the functional outcomes and intraoperative surgeon satisfaction between the fixed-loop and adjustable-loop devices in ACL reconstruction, focusing on the Indian population where economically efficient implant options are a consideration. We hypothesize that intraoperative satisfaction may correlate with final functional outcomes, guiding device selection to optimize patient and surgeon expectations. By providing a comprehensive analysis of intraoperative surgeon satisfaction and two-year postoperative outcomes, this study seeks to address these gaps and provide a deeper understanding of how device selection impacts recovery and functional stability.

## Materials and methods

This retrospective comparative study analyzed patient data from operative and follow-up records to evaluate the effectiveness of fixed-loop and adjustable-loop devices in ACL reconstruction, intraoperative surgeon satisfaction, and final functional outcomes. The study population included 308 patients clinically and radiologically diagnosed with complete ACL tears treated at our hospital between 2017 and 2021. After excluding patients with concurrent ligament injuries, previous knee surgeries, fractures, signs of osteoarthritis, or medical conditions affecting ambulation, 140 patients with a complete two-year follow-up were selected for analysis. Eligible patients were adults (≥18 years) who underwent primary ACL reconstruction with femoral fixation using either a fixed-loop or an adjustable-loop device. Patients were evaluated at six weeks, six months, one year, and two years postoperatively using standardized clinical outcome measures: the Lysholm score, International Knee Documentation Committee (IKDC) score, and Tegner activity score. These validated scales were chosen for their reliability in assessing knee function, stability, and activity level in ACL reconstruction patients [1-3]. Additionally, knee stability, assessed through Lachman and anterior drawer tests, was also collected from records. To address potential confounding factors, we identified variables that could influence the outcomes, such as age, activity level, injury severity, and adherence to rehabilitation protocols. Although adjustments for confounding were not applied in this retrospective study, these variables will be monitored in future prospective studies using propensity score matching or inverse probability weighting to minimize bias.

Surgical technique

After diagnostic arthroscopy, hamstring grafts were harvested from the ipsilateral knee, tripled, and pretensioned to a uniform diameter of 8 mm in all cases. Femoral tunnel drilling was performed through anteromedial and anterolateral portals. The choice of fixation device depended on tunnel length: fixed-loop devices were used if the tunnel length was ≥40 mm, and adjustable-loop devices were selected for tunnels <40 mm. All grafts were positioned to achieve a 20-mm bone-graft interface within the femoral tunnel. Following femoral fixation, the tibial tunnel was drilled using a standard tibial jig, and the tibial end was secured with aperture fixation for both groups. In the adjustable-loop group, grafts were retensioned before final fixation.

Intraoperative assessment included surgeon evaluation of graft tension via palpation and Lachman testing, which was recorded immediately after fixation. Surgeon satisfaction with graft firmness was also documented to explore potential correlations with long-term patient outcomes. To minimize bias in the collection of subjective intraoperative assessments, standardized criteria were used to rate graft firmness and stability, with all surgeons receiving training to ensure consistency in scoring. Intraoperative evaluations were conducted by a limited team of experienced surgeons, further reducing potential variability in assessment. Additionally, the subjective ratings were supplemented with objective measures, such as the Lachman test, to improve the reliability of intraoperative findings. A standardized postoperative rehabilitation protocol was followed for both groups, beginning with static quadricep strengthening exercises and closed chain knee bending from postoperative day 1. Patients started to bear weight as tolerated while using crutches and wearing a knee brace. Knee flexion up to 90° is encouraged by day 12, barring additional procedures like meniscal repair.

Data were analyzed using the Statistical Package for the Social Sciences software (IBM Corp., Armonk, NY). A sample size calculation was conducted to ensure adequate power for detecting clinically meaningful differences between the fixed-loop and adjustable-loop groups. Based on an anticipated effect size of 0.5, a significance level (α) of 0.05, and a desired power of 0.8, it was determined that a minimum of 64 participants per group was required. Therefore, a total of 128 participants was necessary to provide sufficient power to detect statistically significant differences in functional outcomes between the two devices. Continuous variables, such as age, body mass index (BMI), and functional scores, were compared between the fixed and adjustable-loop groups using independent t-tests. In contrast, categorical variables (e.g., stability grades in the Lachman and anterior drawer tests) were analyzed with chi-square tests. Changes in scores over time within each group were assessed using repeated measures analysis of variance. Statistical significance was set at a p value <0.05.

## Results

Among the data collected from 140 patients, 70 patients (46 males) were in the fixed-loop group, and another 70 patients (41 males) were in the adjustable-loop group. The mean age of patients in the fixed-loop group was 26.5 ± 9.5 years, and the mean age in the adjustable-loop group was 29.3 ± 8.7 years. Patients in the fixed- and adjustable-loop groups had a mean injury-to-surgery interval of 0.4 ± 0.5 and 0.5 ± 0.7 months, respectively. Associated meniscal tears were documented in 24 patients in the fixed-loop group and 32 patients in the adjustable-loop group. The mean BMI of patients in the fixed-loop group was 22.1 ± 3; in the adjustable-loop group, it was 24.3 ± 6 (Table 1).

**Table 1:** Patient demographic characteristics for fixed and adjustable-loop groups BMI: body mass index

Feature	Fixed loop	Adjustable loop	p value
Age (years)	26.5 ± 9.5	29.3 ± 8.7	0.06
Total number of subjects	70	70	>0.05
Number of males	46	41	>0.05
Time to surgery (months)	0.4 ± 0.5	0.5 ± 7	0.4
Average follow-up (months)	24	24	>0.05
Meniscal tear	42	44	0.35
BMI (kg/m^2^)	22.1 ± 3	24.3 ± 6	0.12

Stability outcomes were measured using the Lachman and anterior drawer tests at intervals of six weeks, six months, one year, and two years. As shown in Table 2, both device groups demonstrated a high percentage of stability early on, with the adjustable-loop group showing slightly higher stability rates at some time points. However, by the two-year follow-up, both groups had similar stability outcomes, with 49 stable knees in the fixed-loop group and 56 in the adjustable-loop group (p = 0.9). The anterior drawer test findings followed a similar trend, with stability rates that showed no significant differences between the groups at any time point (Table 3).

**Table 2:** Lachman test results for fixed- and adjustable-loop groups over time

Time period	Stability	Fixed loop (number of patients)	Adjustable loop (number of patients)	p value
Six weeks	Stable	57	61	0.5
	Grade 1	11	8	0.6
	Grade 2	2	1	0.8
Six months	Stable	53	58	0.45
	Grade 1	15	11	0.55
	Grade 2	2	1	0.9
One year	Stable	52	56	0.7
	Grade 1	16	13	0.75
	Grade 2	2	1	0.85
Two years	Stable	49	56	0.9
	Grade 1	19	13	0.95
	Grade 2	2	1	0.9

**Table 3:** Anterior drawer test results for fixed- and adjustable-loop groups over time

Time period	Stability	Fixed loop (number of patients)	Adjustable loop (number of patients)
Six weeks	Stable	57	61
	Grade 1	11	8
	Grade 2	2	1
Six months	Stable	53	58
	Grade 1	15	11
	Grade 2	2	1
One year	Stable	52	56
	Grade 1	16	13
	Grade 2	2	1
Two years	Stable	49	56
	Grade 1	19	13
	Grade 2	2	1

Range of motion (ROM) outcomes indicated gradual improvement in both groups, with specific differences noted in knee extension and flexion (Table 4). At six months, the fixed-loop group showed an average extension of 0.1° ± 3.8° compared to -0.2° ± 2.6° in the adjustable-loop group. By the two-year follow-up, the fixed-loop group maintained an extension of 0.5° ± 3.8°, while the adjustable-loop group demonstrated -1.3° ± 3.2° (p = 0.02), indicating a statistically significant difference. Both groups reached comparable levels of flexion improvement at all time points, with the final follow-up showing 137.9° ± 8.3° in the fixed-loop group and 137.8° ± 8.8° in the adjustable-loop group (p = 0.001). Although this p value was low, the clinical relevance of this difference in flexion remains limited due to the minimal difference in mean values.

**Table 4:** Range of motion measurements for fixed- and adjustable-loop groups over time

Time period	Extension			Flexion		
	Fixed loop (°)	Adjustable loop (°)	p value	Fixed loop (°)	Adjustable loop (°)	p value
Preoperative	2.6 ± 6.3	1.7 ± 6	0.34	127.7 ± 27.9	131 ± 32.6	0.034
Six months	0.1 ± 3.8	-0.2 ± 2.6	0.16	136.9 ± 8.2	135.7 ± 8.7	0.029
One year	0.1 ± 2.4	1 ± 2.7	0.06	137.2 ± 7.9	136.6 ± 9.8	0.025
Two years	0.5 ± 3.8	-1.3 ± 3.2	0.02	137.9 ± 8.3	137.8 ± 8.8	0.001

Functional outcomes were assessed using the Lysholm, IKDC, and Tegner activity scores across each follow-up interval (Tables 5, 6). Both groups showed significant improvements in these scores over time. The fixed-loop group's Lysholm score increased from a baseline of 57.78 ± 5.4 to 94.34 ± 8.37 at two years, whereas the adjustable-loop group improved from 52.82 ± 5.3 to 94 ± 7.21. IKDC scores followed a similar trend, with no statistically significant differences at any follow-up point. The Tegner activity scale also showed no significant differences between the groups, with final scores of 5.1 ± 2.1 (fixed loop) and 5.4 ± 2.0 (adjustable loop) at two years.

**Table 5:** Lysholm and IKDC scores for fixed- and adjustable-loop groups over time IKDC: International Knee Document Committee

Score	Loop type	Preoperative	Six weeks	Six months	One year	Two years	p value
Lysholm	Fixed	57.78 ± 5.4	68.34 ± 7.37	79.64 ± 6.4	93.8 ± 9.7	94.34 ± 8.37	0.99
	Adjustable	52.82 ± 5.3	67.47 ± 9.21	79.18 ± 5.7	94.3 ± 6.2	94 ± 7.21	0.18
IKDC	Fixed	38.45 ± 2.8	71.04 ± 1.71	73.12 ± 2.9	82.76 ± 1.8	87.04 ± 1.71	0.99
	Adjustable	33.16 ± 3.64	70.32 ± 2.41	71.94 ± 2.78	83.4 ± 2.7	88.32 ± 2.41	0.17

**Table 6:** Tegner activity scale scores for fixed- and adjustable-loop groups over time

Tegner activity scale	Fixed loop (scores)	Adjustable loop (scores)	p value
Preoperative	6.4 ± 5	6.7 ± 3.6	0.18
Six weeks	3.5 ± 1.9	3.5 ± 2.2	0.58
Six months	3.7 ± 2.8	3.9 ± 1.9	0.12
One year	4.9 ± 3.2	5.1 ± 2.3	0.09
Two years	5.1 ± 2.1	5.4 ± 2	0.21

To evaluate whether surgeon satisfaction with intraoperative graft fixation predicted long-term outcomes, subjective intraoperative assessments of graft firmness and stability were recorded. Surgeon satisfaction with graft firmness was higher in the adjustable-loop group, with 67 cases rated as "firm" versus 57 in the fixed-loop group. The intraoperative Lachman test indicated stable outcomes in 56 cases for the fixed-loop group and 63 cases for the adjustable-loop group, with grade 1 laxity observed in 14 fixed-loop cases and seven adjustable-loop cases (Table 7).

**Table 7:** Surgeons subjective satisfaction with fixed- and adjustable-loop techniques

Parameters	Fixed loop (number of patients)	Adjustable loop (number of patients)
Probe palpation	Firm: 57	Firm: 67
	Lax: 13	Lax: 3
Intraoperative Lachman	Stable: 56	Stable: 63
	Grade 1: 14	Grade 1: 7
Subjective satisfactory fixation	57	67

Despite these intraoperative differences, both groups achieved comparable long-term functional outcomes. Lysholm and IKDC scores showed similar improvements over time, with no statistically significant differences between groups at any follow-up point. These findings suggest that intraoperative surgeon satisfaction did not correlate with final PROs, as both devices demonstrated similar effectiveness in restoring function at two years (Table 5).

Fixed-loop and adjustable-loop devices significantly improved knee stability, ROM, and functional scores over the two-year follow-up. While the adjustable-loop group showed a slight trend toward better knee stability and extension in early follow-ups, these differences did not reach statistical significance at the two-year mark, refuting the hypothesis that device type significantly impacts long-term outcomes. Additionally, intraoperative assessments suggested higher surgeon satisfaction with the adjustable-loop device. However, this satisfaction did not correlate with improved patient outcomes, supporting the need for continued reliance on patient-reported metrics for long-term success.

## Discussion

This study gathered information on the functional outcomes of clinically and radiologically diagnosed ACL tear patients treated with arthroscopic ACL reconstruction using fixed- and adjustable-loop devices. We compared the functional outcomes between the two groups. Additionally, we collected surgeons' intraoperative subjective assessments of graft tension and the final functional outcomes for each patient, an area with limited data in the existing literature. The mean age of patients in our study was 26.5 ± 9.5 years in the fixed-loop group and 29.3 ± 8.7 years in the adjustable-loop group. These ages are similar to those reported by Schützenberger et al. [12], whose study observed a mean age of 26.9 ± 8.7 years (range, 13-50). In the fixed-loop group, 42 (61%) patients had associated meniscal tears, while 44 (64%) patients in the adjustable-loop group had associated meniscal tears. The study by Hagino et al. [13] showed that 186 of 256 (72.7%) acute ACL-torn knees had associated meniscal tears, similar to the findings in the study by Schützenberger et al. [12]. Thus, our study population was comparable to that of existing studies. The inclusion and exclusion criteria ensured a fairly balanced demographic profile between the fixed-loop and adjustable-loop groups. However, slight differences existed, such as a marginally higher mean age and BMI in the adjustable-loop group. Although these differences were not statistically significant, they could influence recovery trajectories and long-term functional outcomes. The slight age difference, for example, may affect healing rates or activity levels, which could subtly influence stability and functional scores. Knee stability, as assessed by the Lachman and anterior drawer tests, showed that the adjustable-loop group had better stability beyond six months than the fixed-loop group. However, the differences were statistically insignificant. Similar statistically insignificant differences in knee stability between the adjustable- and fixed-loop groups were reported by Heng et al. at the two-year follow-up [14]. Likewise, the ROM at the final follow-up was comparable between both groups, with no statistically significant differences.

The functional outcome scores at the final follow-up in our study were consistent with the existing literature. The mean Lysholm score was 94.34 ± 8.37 for the fixed-loop group and 94 ± 7.21 for the adjustable-loop group at the two-year follow-up. Research conducted by Pokharel et al. [15], Lanzetti [3], and Ahn et al. [16] reported mean Lysholm scores of 93.97 for the fixed-loop group and 94.7 for the adjustable-loop group; 92.8 for the fixed-loop group and 93.2 for the adjustable-loop group; and 82.3 for the fixed-loop group and 85.7 for the adjustable-loop group. Similarly, the mean IKDC scores were 87.04 ± 1.71 for the fixed-loop group and 88.32 ± 2.41 for the adjustable-loop group. Studies by Pokharel et al. [15], Lanzetti [3], and Ahn et al. [16] reported mean IKDC scores of 82.52 for the fixed-loop group and 83.98 for the adjustable-loop group, 89.5 for the fixed-loop group and 90.4 for the adjustable-loop group, and 79.43 for the fixed-loop group and 78.6 for the adjustable-loop group, respectively. The mean Tegner activity scores were 5.1 ± 2.1 for the fixed-loop group and 5.4 ± 2.0 for the adjustable-loop group, similar to the scores reported by Heng et al. [14]. A systematic review and meta-analysis by Elmholt et al. indicated that adjustable and fixed loops showed similar functional outcomes, though with low-quality evidence [17]. These findings, along with our results, suggest that the clinical functional outcomes of fixed and adjustable loops are similar. For most orthopedic procedures, intraoperative feedback is believed to correlate with the final patient outcome. We explored the surgeons' subjective satisfaction immediately after graft fixation, though limitations exist due to interobserver and intraobserver variabilities. Interobserver variability was minimized as the same team of two arthroscopic surgeons operated on all selected patients. The surgeons' subjective satisfaction immediately after fixation was higher in the adjustable loop group (p < 0.05). However, at the final follow-up, the surgeons' subjective satisfaction did not correlate with the functional outcomes, as both the fixed- and adjustable-loop groups demonstrated similar functional outcomes at the two-year follow-up.

The lack of significant differences in functional outcomes between fixed and adjustable-loop devices may indicate that both devices meet the minimum biomechanical stability required for ACL reconstruction, especially under typical loads experienced in daily activities. Additionally, individual patient factors such as adherence to physical therapy and variations in healing responses might mitigate small device-specific differences. For clinical practice, this suggests that the choice of device may be secondary to postoperative rehabilitation and patient engagement in recovery, highlighting the importance of optimizing these factors to improve long-term outcomes.

Limitations of the study

While the study provides valuable insights into comparing fixed- and adjustable-loop femoral suspensory devices in ACL reconstruction, several limitations should be acknowledged. The retrospective nature of the study might introduce selection bias, as the patients included may not represent the entire population undergoing ACL reconstruction. The accuracy and completeness of data collected from the operation theater and follow-up records may vary, potentially affecting the reliability of the findings. Differences in patient activity levels after surgery, individual variations in surgeon technique, and patient-specific health factors like comorbidities or prior knee injuries may have influenced outcomes independently of device type. These factors were not fully accounted for in this study, potentially impacting the generalizability of the findings. Future prospective studies with rigorous control for these confounders are needed to validate these results. A larger sample size would increase the statistical power of the study, allowing for more definitive conclusions. Since this is a single-center study, the findings may not be generalizable to other populations or healthcare settings, as results may be influenced by specific surgical techniques, rehabilitation protocols, and patient characteristics prevalent at the study center. The subjective nature of assessments such as the Lachman and anterior drawer tests and surgeons' subjective satisfaction can introduce variability between examiners, potentially affecting the accuracy of the results. While PROs provide valuable information, they may be influenced by factors such as pain tolerance, expectations, and psychological factors. A longer follow-up period would provide more comprehensive information about the long-term durability of the grafts and the stability of the reconstructed ACL.

## Conclusions

This study reinforces prior findings that both fixed- and adjustable-loop devices yield similar functional outcomes in ACL reconstruction, as evidenced by comparable Lysholm, IKDC, and Tegner activity scores over two years. Additionally, it highlights that intraoperative surgeon satisfaction with graft firmness in adjustable loops does not necessarily predict long-term functional success. These insights add depth to the body of knowledge on ACL reconstruction device efficacy, suggesting that PROs should continue as a primary metric for assessing success. Future research should focus on larger, multicenter studies to enhance the generalizability of these findings. Clinically, this study supports using either device type based on availability and surgeon preference, as both options provide effective outcomes for patients.
